# Spatial modeling of personalized exposure dynamics: the case of pesticide use in small-scale agricultural production landscapes of the developing world

**DOI:** 10.1186/1476-072X-8-17

**Published:** 2009-03-30

**Authors:** Stefan Leyk, Claudia R Binder, John R Nuckols

**Affiliations:** 1 Department of Geography, University of Colorado, 260 UCB, Boulder, CO 80309, USA; 2 Department of Geography, University of Zurich, Winterthurerstrasse 190, CH-8057 Zurich, Switzerland; 3 Department of Environmental and Radiological Health Sciences, Colorado State University, 1681 Campus Delivery, Fort Collins, CO 80523, USA; 4 Division of International Epidemiology and Population Studies, Fogarty International Center, National Institutes of Health, Bethesda, MD 20892, USA

## Abstract

**Background:**

Pesticide poisoning is a global health issue with the largest impacts in the developing countries where residential and small-scale agricultural areas are often integrated and pesticides sprayed manually. To reduce health risks from pesticide exposure approaches for personalized exposure assessment (PEA) are needed. We present a conceptual framework to develop a spatial individual-based model (IBM) prototype for assessing potential exposure of farm-workers conducting small-scale agricultural production, which accounts for a considerable portion of global food crop production. Our approach accounts for dynamics in the contaminant distributions in the environment, as well as patterns of movement and activities performed on an individual level under different safety scenarios. We demonstrate a first prototype using data from a study area in a rural part of Colombia, South America.

**Results:**

Different safety scenarios of PEA were run by including weighting schemes for activities performed under different safety conditions. We examined the sensitivity of individual exposure estimates to varying patterns of pesticide application and varying individual patterns of movement. This resulted in a considerable variation in estimates of magnitude, frequency and duration of exposure over the model runs for each individual as well as between individuals. These findings indicate the influence of patterns of pesticide application, individual spatial patterns of movement as well as safety conditions on personalized exposure in the agricultural production landscape that is the focus of our research.

**Conclusion:**

This approach represents a conceptual framework for developing individual based models to carry out PEA in small-scale agricultural settings in the developing world based on individual patterns of movement, safety conditions, and dynamic contaminant distributions.

The results of our analysis indicate our prototype model is sufficiently sensitive to differentiate and quantify the influence of individual patterns of movement and decision-based pesticide management activities on potential exposure. This approach represents a framework for further understanding the contribution of agricultural pesticide use to exposure in the small-scale agricultural production landscape of many developing countries, and could be useful to evaluate public health intervention strategies to reduce risks to farm-workers and their families. Further research is needed to fully develop an operational version of the model.

## Background

The importance of reliably assessing human exposure to environmental toxins from agricultural management activities has been growing due to human health impairments within farm-workers and the rural population, worldwide [1], and particularly in developing countries [2]. According to estimations of The World Bank there are 355,000 deaths each year due to unintentional poisoning from pesticide exposure [3]. Despite recognized assessment uncertainties [4] the majority of these incidents are clearly related to developing countries in Africa, Asia and Central and South America [5,6]. One common management practice in these regions is small-scale farming with manual (backpack) pesticide spraying. The main reasons for pesticide exposure in such agricultural production landscapes are the availability of toxic substances, as well as a lack of protective measures, education and health care [7]. Educational efforts to reduce health risks of farmers in these regions have had limited success [2,6]. The lack of quantitative information with regard to potential exposure pathways to estimate the risks for different groups, such as inhabitants or backpack sprayers could be a factor.

Exposure occurs when there is contact of a chemical, physical or biological agent of a specific concentration with an organism for an interval of time [8,9]. Human exposure to pesticides can occur through inhalation via air or dust, dermal contact with the pesticide or deposited residue on surfaces, ingestion, or interpersonal contact with adherent residues on the body (especially hands) or clothing. Personal activity patterns were recognized as one of the main determinants of the magnitude, frequency, duration, and pathways of exposure [10]. Thus a need exists for improving exposure assessments on the individual level resulting in personalized exposure assessment (PEA) [11]. To date there is no model approach that completely implements the conceptual idea of PEA in small-scale agriculture in the developing world.

Spatial factors such as the location of the exposed individuals and their activity in relation to the contaminant source have been identified as important determinants for more reliable exposure assessments [12]. Thus new technologies that allow the assessment of external environmental exposure to support PEA such as Geographical Information Systems (GIS) and environmental sensors have been discussed [11,13,14]. Case studies for exposure assessment based on GIS are reported for pesticides [15,16], urban pollution [17,18], trichloroethylene in water [19], and pollutants from landfill sites [20]. Whereas these approaches resulted in the improvement of exposure assessments, impediments to a specific personalized analysis have been encountered. Identified impediments include the high aggregation of spatial data, e.g., land use records [21], the scale dependence of exposure estimates [22], the lack of consideration of spatial and temporal variation [23] and the lack of accounting for individual activity patterns [24].

In response to some of these impediments Spatio-Temporal Information Systems (STIS) [25,26] were developed to build up individual histories of exposure to arsenic concentrations in water supplies. These approaches include environmental variations and residential mobility history of individuals. However, STIS do not incorporate personal activity patterns, which are highly relevant to pesticide exposure.

The first approaches that incorporated personal activity patterns to break down the population level to the individual level for deriving exposure metrics [11] applied Global Positioning Systems (GPS) [27,28]. Here, individual time-location data and activity-related information relevant for exposure were collected. However, it remains an open question how to incorporate such activity data into a model framework that links them with spatio-temporal distributions of contaminant concentrations in the environment for PEA [29,30].

Spatial-explicit dynamic modeling approaches such as individual-based models (IBM), which are a subcategory of agent-based models (ABM), have gained increased attention for epidemiological studies and public health [31,32]. IBMs have been applied to model management decisions in farming systems [33,34], human-wildlife interactions [35], as well as integrated pest management [36]. By modeling activity and characteristics of moving individuals, IBMs break down the analysis to the individual level [37]. IBMs thus account for heterogeneity within the population with regard to individual characteristics and for local interactions between individuals and the environment [38,39]. One common approach of modeling actors or individuals in the physical environment is to couple ABMs with Cellular automata (CA) [40]. CAs create a discrete time system in a spatial context [41] in which cells in a lattice undergo state transitions at a given time interval. These transitions are defined by simple rules of interactions between cells within the local environment [42,43]. These conceptual principles illustrate that IBMs provide an appropriate methodological framework to address the problem of PEA. However, IBMs have never been applied for developing spatially explicit PEA approaches.

In this paper we propose a conceptual framework for the assessment of personalized pesticide exposure of farm-workers due to primary drift and dermal contact with deposited contaminant residuals in small-scale agricultural management settings in less-developed regions where backpack spraying is carried out. The proposed IBM prototype integrates dynamic distributions of deposited contaminant residuals, which are influenced by decisions related to pesticide application, with individual spatial activity of farm-workers under different safety conditions. The model prototype incorporates simulated movement patterns and simplified assumptions for the dynamics in the system. The main purpose of this conceptual framework is to demonstrate the capability of such tools to assess effects of protection measures, activities performed, patterns of movement as well as patterns of pesticide application on potential exposure of individuals. We develop this conceptual model and test first simulations using underlying spatial data of Vereda la Hoya, a rural part of the Departamento de Boyacá, Colombia.

## Methods

### Study area and Data

The study area, Vereda la Hoya, is located in a less-developed region in the rural parts of the Departamento de Boyacá, Colombia, in the eastern chain of the Andes. The region is characterized by a high degree of integration between residential and agricultural land, cultivation of potatoes and carrots on small parcels (minifundios) and a low level of technological development. In response to major pests in the area different insecticides and fungicides are frequently applied throughout the vegetation period using manual backpacking sprayers under low safety conditions [44]. Health impacts including headache, nausea, and blurred vision, as a consequence of both occupational and non-occupational exposure to pesticides are documented for comparable study areas [45].

Spatial datasets used to build up the model environment included land use raster data of resolution 15 m derived from Landsat ETM+ satellite interpretations of the year 2000, as well as residence and management parcel maps all obtained from local authorities. We created artificial individuals and defined their hypothetical patterns of movement by simulating locations for each time step, as well as a very simplified set of activities performed that would be based on field observations [44].

### Toolkit

For the implementation of the exposure model, we used the Recursive Porous Agent Simulation Toolkit (RePast) [46]. RePastJ contains a set of Java libraries allowing for efficient and quick adoption of the RePast platform to build an agent-based model and is coupled with GIS software to make use of spatial analysis tools during simulations. We extended the set of existing libraries to develop an individual-based model prototype which links a dynamic cell-based environment (environmental subsystem in which the cell is the modelling unit) with individuals that are defined by patterns of movement, activity and protection measures (social subsystem).

### Conceptual framework

**Figure 1:**
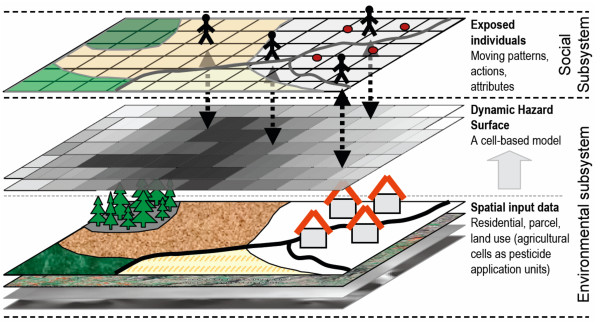
**The conceptual framework of the PEA model**. The individuals of the social subsystem move within and interact with, the environmental subsystem in which the hazard surface is computed.

In short, exposure occurs if a person is at a location where there is some contaminant concentration in the environment and if there is a contact between the human body and the contaminant e.g., caused by a particular activity. The intensity of exposure of one individual can vary depending on attributes that indicate safety measures taken or protection equipment used [45] but also on frequency and duration of contact with the active ingredient [47]. Consequently, the assessment of individual exposure to pesticides requires an individual-based framework, which incorporates a social and an environmental component, as well as defined parameters and decision rules. Our model includes interactions between moving individuals [27,35] and the dynamic hazard distribution in the environment to estimate individual potential exposure (Figure 1). The conceptual framework incorporates some simplifications to reduce the complexity of the system and to focus on the methodological steps and implementation mechanisms relevant for PEA:

- Farm-workers are the only target group for PEA; only a small number of individuals are defined by a set of activities and model parameters for different safety scenarios. This allows the evaluation on the individual level, the direct comparison between individuals and the analysis of effects of patterns of movement and hazard dynamics on PEA.

- Because of a lack of environmental field measurements and limited knowledge of pesticide deposition curves [23,48] the hazard values are computed as relative degrees between 0 and 1 instead of using concentrations of the active ingredient, environmental or climatic conditions.

- Exposure due to primary drift of pesticides at the time of pesticide application, and dermal contacts with contaminated plant material and soil while working and moving in the area of pesticide application are considered only. These represent significant pathways for external exposure of the study population that is farm-workers, and thus a suitable case for incorporating environmental and social dynamics for PEA.

### Dynamic hazard surfaces in the environmental subsystem

**Figure 2:**
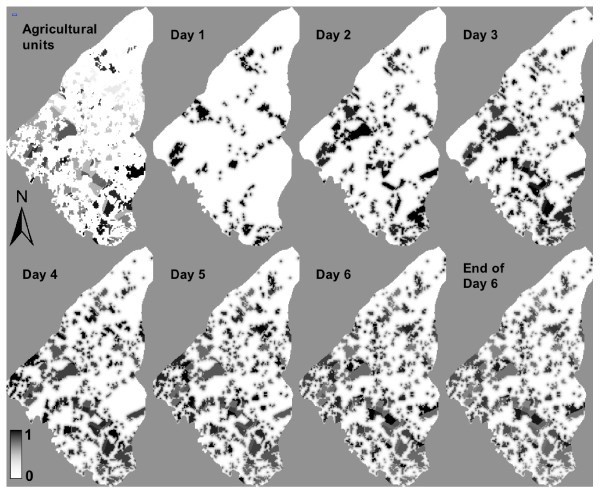
**Computation of dynamic hazard surfaces**. Relative hazard values are computed throughout the model period for one scenario of pesticide application at the beginning of each day.

We use the dynamic cell-based component to represent the ***environmental subsystem***, which essentially represents location of potential exposure to pesticides based on land use and pesticide distributions after the methods described by other studies [15,16,21,48-51]. Locations of pesticide application at the beginning of each day as well as application frequency, are determined to simulate management decision rules for the agricultural setting of our study area [44]. *Land use *underlies short-term and long-term dynamics that impact exposure and are related to decision-making of the farmers. Here the short-term dynamics related to seasonal agricultural management are addressed to label the location of crops sprayed on each day, resulting in a dynamic surface of cell values that indicate hazard degrees (Figures 1, 2).

#### Initial hazard distribution

To simulate pesticide application activity a set of agricultural fields is selected randomly to apply pesticides to an equal number of field IDs at the beginning of each day, excepting Sunday, resulting in specific patterns of pesticide application (Figure 2). Each agricultural field is sprayed once a week, over a period of 12 weeks twice a year, which is a simplification of the common practice in the study region [44,52]. Typically, different ingredients are used and varying application rates are applied in different stages of crop phenology. In our model prototype, a relative hazard value *H→[0,1] *is set to 1.0 at the locations of pesticide application, instead of assuming specific concentrations of the active ingredient. However, our model is designed to allow the definition of *H *values of the individual cells as a function of concentration and application methods for varying active ingredients. To account for spatial and classification uncertainty in the land use data but also for some potential effects of secondary drift after application, the values of the non-agricultural cells in the neighborhood of the cells sprayed are defined by proportionally decreased hazard values in order to reflect reduction in pesticide concentration of drift as a function of drift distance [48]. In our prototype model, we used a simplified rule, which reduces the hazard values *H*_*j *_at location *j *by 50% per 15 m distance according to the cell size (**Equation 1**):

(1)

where *jj *indicates the location of one of the eight direct neighbors and *d *indicates an adjustment factor for the distance between the considered cell and its neighbor (*d *= 1 for 4-connected, *d *= 1.41 for diagonal 8-connected neighbors). As Equation 1 demonstrates *H*_*j *_is the maximum value of the eight neighboring hazard values.

#### Dynamics in the hazard surface

For each location *j *and each time step *t*, which covers 30 min, the decay of the hazard values *H*_*j, t *_over time is implemented as the degradation half-life to account for pesticide persistence [53] (Equation 2):

(2)

where *t*_0 _indicates the first time step when pesticide application takes place and the number 32 indicates the number of time steps per day. *Halflife *is the estimated time period for reduction of the concentration of the active pesticide ingredient at the time of spraying to degrade to one-half that concentration under field conditions. We exemplarily used a half-life value of 10 days. Pesticide application at the beginning of the next day initiates a new hazard surface, which is overlaid with the first one. A maximum rule is applied to this overlay operation to compute the new value *H*_*j, t *_so that the model accounts for the dynamics of addition and degradation of the active ingredient in the hazard surface more realistically.

### The individual-based component within the social subsystem

**Figure 3:**
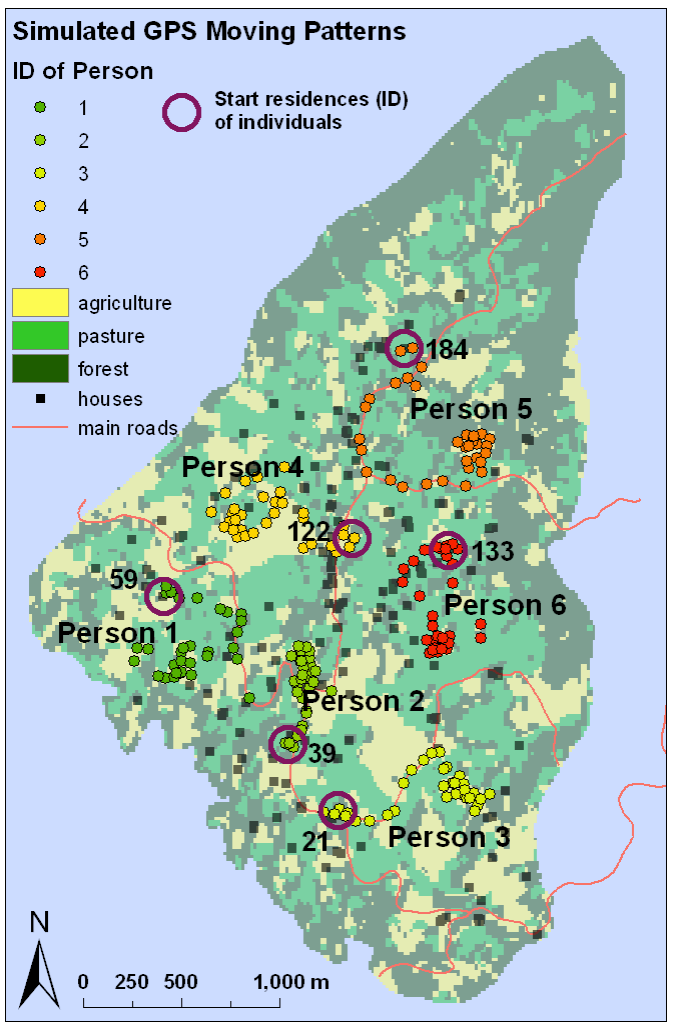
**Synthetic spatial patterns of movement of individuals**. Illustration of patterns of movement and home residences of individuals (purple circles).

The individual-based component of the conceptual framework is implemented in the ***social subsystem ***[38,54]. Individual patterns of movement and activity performed during the day are defined to simulate individual spatial activity after methods described in other studies [27,28,30,31,53,55,56] (Figure 3). We defined individual weights for specific activities performed as well as safety conditions [45] for these activities.

The model is fed with recurring daily patterns of movement of six individual farm-workers. We artificially simulated locations of individuals that would have been surveyed using GPS during the day (Figure 3) [27,30]. Every day individuals perform a number of activities [11,28], i.e., being at their home residences, traveling to the agricultural field, working at the field (weeding, drilling, plugging), traveling back to residences, and spending time at home or nearby residences, etc. (Table 1). In order to evaluate the influence of each activity performed on potential exposure we included activity-related weights *A→[0,1] *in Equation 3. Different activities can be related to different degrees of potential exposure i.e., higher weights are included for activities that are related to agricultural work than for being at home or traveling. In addition, potential occupational exposure depends on safety related determinants i.e., work practice and type of clothing including gloves or masks [44,53,55]. To simulate scenarios of low and high individual safety we varied the activity-related weights. For the same activities we included higher weights under low safety and lower weights for high safety scenarios (Table 1). The values of the weights are relative scores, which do not reflect measurements. They mainly serve the purpose of weighting the different activities against each other for varying safety conditions (Table 1). The ranking between activities remains the same for both safety scenarios (Table 1).

**Table 1:** Activity-related weighting scheme to carry out PEA for different safety scenarios

**Activity at time steps (periods) modeled**	**Low safety**	**High safety**	**Justifications for weights in different safety scenarios (impact of activity on exposure)**
At home/nearby (t_1_–t_4_)	**0.4**	**0.3**	Frequent contacts with surfaces without protection but outside fields (lower H values). Higher risk "perception" in high-safety scenario.

Traveling to field (t_5_–t_6_)	**0.3**	**0.2**	Uptake at shoes/clothes possible but mostly outside fields. Different clothes/shoes for work and at home in high-safety scenario.

Working on the field (t_7_–t_23_)	**1**	**0.6**	Frequent dermal contacts and inhalation (primary drift) on the field; **Low safety scenario**: no protection equipment (same clothes at home and for work, no gloves, masks, glasses, or coveralls); **High safety scenario**: Different clothes for work and at home, and "some" protection equipment

Traveling back (t_24_–t_25_)	**0.3**	**0.2**	See traveling above

At home/nearby (t_26_–t_32_)	**0.4**	**0.3**	See being at home above

t_#1_–t_#2_: Period (number of time steps) in which activity is performed and thus the weights in the same row are used for different safety scenarios.

### Personalized exposure assessment

The environmental and social subsystems are linked after methods described in the literature [25,26,29,32] to define system behavior [40,57], and estimate personal exposure for each individual using a defined temporal resolution [47].

#### Modeling schedule

At the beginning of each of the six working days during the modeling period, we simulated pesticide application and computed the initial hazard surface (Equation 1). Each location visited by the individuals represents the "average" position between two time steps. In our prototype model we use a time step of 30 minutes. Individuals visit 32 locations during 16 hours daily (Figure 3) summing up to 192 time steps for 6 days. With each time step the hazard surface values change, synchronously, based on Equation 2. Nighttime is embedded as a shift in Equation 2 to decrease the hazard values according to an 8 hour time period before the pesticide application begins the next day.

#### Computing the exposure value

At each position *j*, which is visited by an individual at time step *t*, the value of the underlying hazard surface *H*_*j, t *_(Equations 1,2) is recorded. The potential external exposure *E*_*n, j, t *_of individual *n *at location *j *for time step *t *is then estimated using Equation 3:

(3) *E*_*n, j, t *_= *A*_n, j, t _*H_*j, t*_

where *A*_*n, j, t*_→*[0,1] *is the weight related to the activity performed by individual *n *at time step *t *under the considered safety level. Thus the values of *A*_*n, j, t *_change over time depending on the activity performed at time step *t *and the safety level assumed for this individual *n *(Table 1). The values *E*_*n, j, t *_could be expressed as concentrations if field measurements were available since they are derived from *H*_*j, t *_values. By translating *H*_*j, t *_directly into *E*_*n, j, t *_without weighting (*E*_*n, j, t *_= *H*_*j, t*_) PEA is based on the dynamic hazard distribution, which results from patterns of pesticide application and individual patterns of movement only.

#### Evaluation and assessment

**Figure 4:**
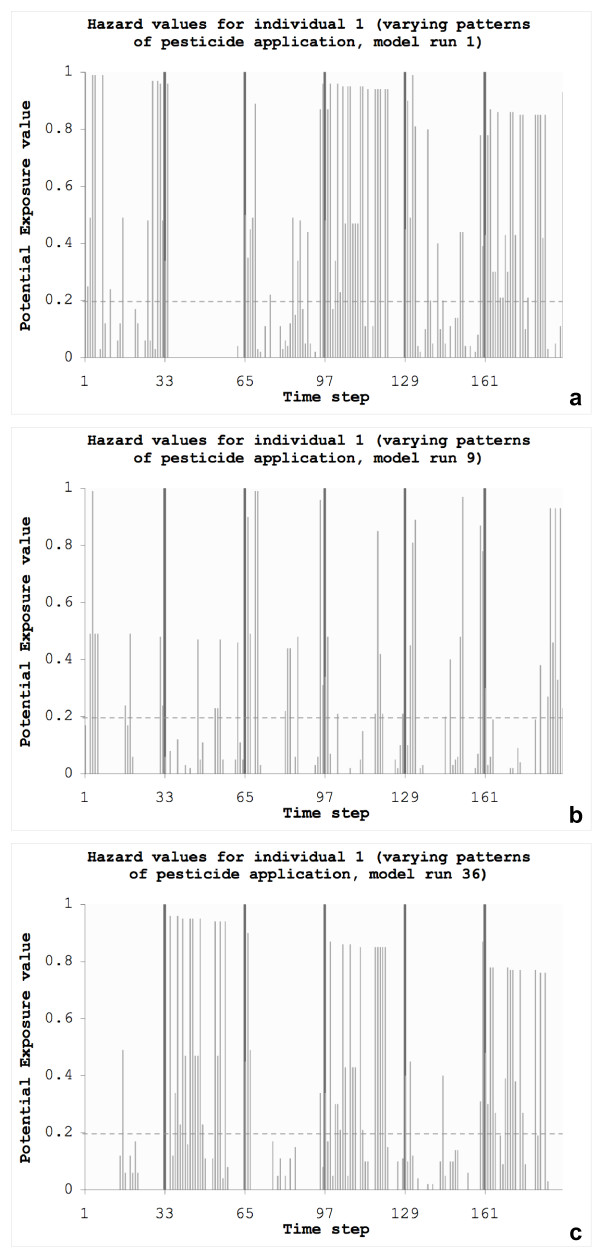
**Extracting potential exposure values for varying patterns of pesticide application**. Hazard values recorded for individuals that show the same patterns of movement for varying patterns of pesticide application over the model period (192 time steps): (a) model run 1, (b) model run 9 and (c) model run 36. The dashed line indicates the threshold value *R*^*E *^(*R*^*E *^= 0.2) for computing frequency and duration. Vertical lines separate days (32 time steps).

In the model prototype, the exposure values *E*_*n, j, t *_are recorded for each individual as time series data over the modeling period (Figure 4) and can be exported for further evaluation. We derived the magnitude , which is used to normalize the exposure values to a potential average concentration value per time step over the model period (Equation 4):

(4)

where *T*^*Tot *^is the number of time steps in the model period (*T*^*Tot *^= 192 for 30 min temporal resolution) and *C *is the number of exposure estimates recorded.

Our simulation model also derives *frequency *and *duration *of potential external exposure. Frequency  is defined as the number of time steps during the modeling period where the underlying hazard value of the visited cell exceeds a user-specified threshold *R*^*E *^divided by the total number of time steps during the model period. Duration  is defined as the maximum number of time steps on one day where the underlying hazard value of the visited cell exceeds a user-specified threshold value *R*^*E*^, subsequently, divided by the total number of time steps during one day. The computation of  is terminated if the hazard values of the visited locations are below *R*^*E *^for at least four time steps, subsequently, which is an arbitrary threshold value. Computing  is reinitiated after the next cell is visited with an underlying hazard value exceeding *R*^*E*^. The measures  and  are computed as follows:

(5)

and

(6)

with

(7)

where *R*^*E *^represents the user-specified hazard threshold value, *T*^*Tot *^indicates the number of time steps during the model period (*T*^*Tot *^= 192 steps), *T*^*Day *^is the number of time steps in one day, and  is the duration or the number of time steps where the visited hazard cell values are below *R*^*E*^, subsequently. is expressed as a unit-less relative count *→[0,1]*, where 0 indicates zero time steps and 1 indicates 192 time steps;  with *→[0,1] *can be transferred to time units as derived from the number of time steps where 0 equals zero time units and 1 represents 32 time steps (16 h).

### Scenario simulation and sensitivity analysis

We tested the sensitivity of the exposure estimates to both varying individual patterns of movement [11,27] and varying patterns of pesticide application, which result in different hazard distributions in the environment [23]. To evaluate the effect of patterns of movement on potential exposure we run the model 100 times for the same patterns of pesticide application but varying patterns of movement. We repeated these model runs for the "non-weighted scenario", as well as for both the "low safety" and "high safety" scenarios. The locations to be visited by the individuals at each time step were randomly determined within a circular neighborhood with radius 90 m and the originally defined coordinates as center points. This resulted in deviating individual patterns of movement that still remained similar to the original path defined. The use of radius of 90 m is hypothetical to demonstrate the feasibility of the modeling approach. It indicates how much the daily patterns of movement of the same individual could deviate due to different circumstances that cause activities at other locations.

To examine the sensitivity of the exposure estimates to changes in decisions related to pesticide application we run the model another 100 times for the same patterns of movement but varying patterns of pesticide application (Figure 4a, b and 4c show three examples) and repeated this for the "non-weighted scenario" as well as for the two safety scenarios. In each model run the sets of agricultural parcels that are sprayed at the beginning of each day are randomly determined such that each parcel ID is sprayed once during the model period. This results in very different dynamic hazard distributions for every model run i.e., the same cells can have different values at the same time step depending on the schedule for pesticide application.

We used the R statistical package [58] to derive summary statistics for the extracted values magnitude , frequency  and duration  of each individual over the model runs including standard deviations, 1^st ^and 3^rd ^quartiles, minima, maxima, medians and means ,  and . These summary statistics describe the variations in exposure estimates over the model runs for each individual and thus provide an indication of the influence of individual patterns of movement as well as patterns of pesticide application on PEA. Lower confidence limits (LCL) and upper confidence limits (UCL) of the means ,  and  (confidence level 95%) are calculated to show how much uncertainty there is in our estimates of the true means for each individual.

## Results

The results of the PEA for six individuals are presented for varying patterns of pesticide application and constant patterns of movement in Table 2 and for varying patterns of movement and constant patterns of pesticide application in Table 3. The means of magnitude , frequency  and duration  are given for all three scenarios "non-weighted", "low safety" and "high safety". For the non-weighted scenario the lower (LCL) and upper confidence limits (UCL) of these means (confidence level 95%) are given.

**Table 2:** Results of PEA scenarios (100 model runs each) for changing patterns of pesticide application

**Individual**	**Non-weighted**						**Low safety**			**High safety**		
(residence)												
**1 (59)**	**0.21**	0.23 0.19	**0.33**	0.36 0.29	**0.56**	0.62 0.49	**0.18**	**0.30**	**0.50**	**0.12**	**0.22**	**0.38**

**2 (39)**	**0.20**	0.23 0.18	**0.30**	0.33 0.26	**0.53**	0.60 0.46	**0.18**	**0.27**	**0.47**	**0.12**	**0.21**	**0.36**

**3 (21)**	**0.22**	0.26 0.19	**0.34**	0.39 0.29	**0.55**	0.64 0.47	**0.19**	**0.31**	**0.52**	**0.13**	**0.26**	**0.44**

**4 (122)**	**0.30**	0.33 0.27	**0.42**	0.47 0.38	**0.71**	0.78 0.64	**0.26**	**0.39**	**0.67**	**0.18**	**0.32**	**0.57**

**5 (184)**	**0.14**	0.16 0.12	**0.20**	0.22 0.17	**0.32**	0.38 0.27	**0.12**	**0.18**	**0.29**	**0.08**	**0.14**	**0.23**

**6 (133)**	**0.10**	0.12 0.08	**0.14**	0.17 0.11	**0.32**	0.40 0.23	**0.08**	**0.12**	**0.30**	**0.06**	**0.10**	**0.25**

= mean of exposure magnitudes,  = mean of frequency values (proportional count; 1.0 = 192 time steps),  = mean of duration values (proportional count of subsequent time steps; 1.0 = 32 time steps). In the scenario "non-weighted" lower confidence limits (LCL; subscripted) and upper confidence limits (UCL; superscripted) of the means (95% confidence level) are given; these ranges would be comparable for the means in both safety scenarios.

**Table 3:** Results of PEA scenarios (100 model runs each) for varying individual patterns of movement

**Individual**	**Non-weighted**						**Low safety**			**High safety**		
(residence)												
**1 (59)**	**0.25**	0.25 0.24	**0.37**	0.38 0.37	**0.60**	0.62 0.58	**0.21**	**0.33**	**0.55**	**0.14**	**0.28**	**0.42**

**2 (39)**	**0.28**	0.29 0.28	**0.41**	0.42 0.41	**0.57**	0.59 0.55	**0.24**	**0.38**	**0.52**	**0.17**	**0.31**	**0.42**

**3 (21)**	**0.31**	0.31 0.30	**0.47**	0.47 0.46	**0.76**	0.77 0.75	**0.27**	**0.42**	**0.69**	**0.18**	**0.33**	**0.53**

**4 (122)**	**0.35**	0.35 0.34	**0.53**	0.53 0.52	**0.78**	0.79 0.76	**0.29**	**0.48**	**0.69**	**0.20**	**0.38**	**0.56**

**5 (184)**	**0.24**	0.24 0.23	**0.38**	0.38 0.37	**0.47**	0.49 0.45	**0.22**	**0.34**	**0.41**	**0.15**	**0.27**	**0.32**

**6 (133)**	**0.13**	0.13 0.12	**0.20**	0.20 0.19	**0.31**	0.33 0.30	**0.11**	**0.17**	**0.28**	**0.08**	**0.13**	**0.20**

= mean of exposure magnitudes,  = mean of frequency values (proportional count; 1.0 = 192 time steps),  = mean of duration values (proportional count of subsequent time steps; 1.0 = 32 time steps). In the scenario "non-weighted" lower confidence limits (LCL; subscripted) and upper confidence limits (UCL; superscripted) of the means (95% confidence level) are given; these ranges would be comparable for the means in both safety scenarios.

### PEA using the non-weighted hazard values

**Figure 5:**
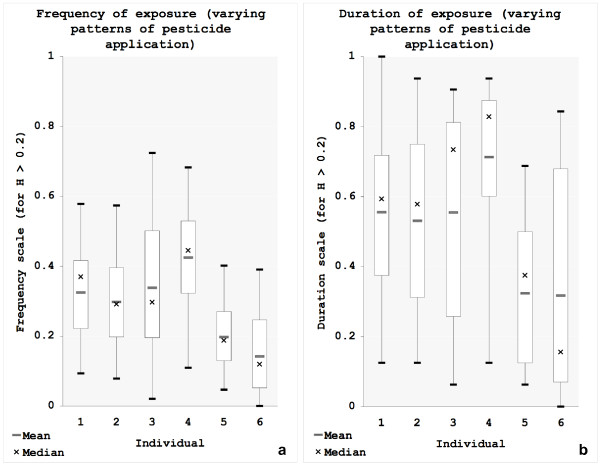
**Results of PEA for non-weighted hazard values and varying patterns of pesticide application**. Summary statistics in boxplots (min, max, 1^st ^and 3^rd ^quartile, mean and median for (a) Frequency measures and (b) Duration measures.

The results of the PEA based on non-weighted hazard values illustrate the differences in potential exposure between the six individuals for varying patterns of pesticide application (Table 2) and varying patterns of movement (Table 3) without accounting for activities or safety conditions. For varying patterns of pesticide application there is a high variation in intra- and inter-personal estimates over the model runs (Table 2): The mean of exposure magnitudes  ranges from 0.10 for person 6 (LCL = 0.08; UCL = 0.12) to 0.30 for person 4 (LCL = 0.27; UCL = 0.33), which are proportions of the maximum concentrations indicated as 1.0 at the field units; the mean of frequency values  ranges from 0.14 (or 27 time steps; LCL = 21 time steps; UCL = 33 time steps) for person 6 to 0.42 (or 81 time steps; LCL = 69; UCL = 90) for person 4; the mean of duration values  ranges from 0.32 (or 10 time steps, subsequently; LCL = 7; UCL = 13) for person 6 to 0.71 (or 23 time steps, subsequently; LCL = 20; UCL = 25) for person 4. As can be seen LCL and UCL define wide ranges of estimates. This means that the individual model runs can result in very different rankings of the individuals illustrating the strong influence of the patterns of pesticide application resulting in different dynamic hazard distributions. This high degree of variation in estimating exposure is also illustrated in Figure 5a and 5b, which show the boxplots for the recorded values  and  over the model runs.

**Figure 6:**
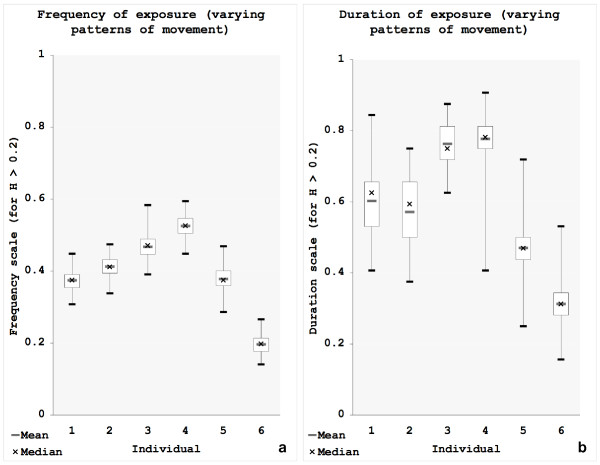
**Results of PEA for non-weighted hazard values and varying patterns of movement**. Summary statistics in boxplots (min, max, 1^st ^and 3^rd ^quartile, mean and median for (a) Frequency measures and (b) Duration measures.

Varying patterns of movement result in less variation in exposure estimates than varying patterns of pesticide application as depicted in Figure 6a and 6b and Table 3:  ranges from 0.13 for person 6 (LCL = 0.12; UCL = 0.13) to 0.35 for person 4 (LCL = 0.34; UCL = 0.35;  ranges from 0.20 (or 38 time steps; LCL = 36; UCL = 38) for person 6 to 0.53 (or 102 time steps; LCL = 100; UCL = 103) for person 4;  ranges from 0.31 (or 10 time steps, subsequently; LCL = 10; UCL = 11) for person 6 to 0.78 (or 25 time steps, subsequently; LCL = 24; UCL = 25) for person 4. The smaller ranges defined by LCL and UCL indicate less uncertainty in estimating the true means for varying patterns of movement than for varying patterns of pesticide application and thus result in fewer changes in the rankings of individuals over the model runs. Nevertheless the boxplots for  (Figure 6a) show a higher variation than for  (Figure 6b).

### PEA including activities for different safety levels

The inclusion of activity-related weights for low and high safety scenarios results in similar exposure patterns but shows a systematic shift of the estimates due to the weighting procedure. Nevertheless there are some changes in the rankings between individuals. At the low safety scenario for varying patterns of pesticide application (Table 2) it can be observed that individual 1 and 3 switch the ranks for  and that individual 5 and 6 have slightly different values  whereas they showed the same value for the non-weighted scenario (Table 2). These changes demonstrate that the weighting of hazard values at visited locations based on activity and safety level has some influence on PEA. Because of the described high variation in exposure estimates for varying patterns of pesticide application these influences have a minor effect.

More changes can be observed in the model runs for varying patterns of movement (Table 3). For example at the high safety scenario individuals 1 and 5 switch ranks for ; individuals 3 and 4 show different values  whereas they had the same value at the low safety scenario. Because of the lower variation in exposure estimates for varying patterns of movement than for varying patterns of pesticide application the safety-related weighting results in more visible effects.

## Discussion

### Benefits

The presented framework follows the conceptual ideas of PEA as elaborated in the recent literature [11,12]. The approach refers to the described agricultural production landscape of small-scale farming systems where backpack pesticide spraying is carried out – a practice with a global extent and thus a serious issue for public health [7]. This paper demonstrates how personal activity data surveyed using GPS [27], questionnaires and observations can be integrated with a dynamic individual-based model framework for PEA to break down the level of analysis to the individual level. For each time step, potential exposure of individuals is estimated based on the hazard surface value at their current position, as well as activities performed under low and high safety conditions. The presented results demonstrate the framework allows to study the effects of agricultural management such as pesticide application, which determines the dynamic hazard distribution, individual patterns of movement as well as activities under different safety conditions on individual exposure estimates. These results demonstrate the suitability of the approach for a more realistic risk assessment. The direct benefits from this conceptual effort are:

(i) A framework for PEA, which incorporates individual patterns of movement, individual activities and safety conditions as well as dynamics in the hazard value distribution

(ii) The computation of composite exposure metrics (magnitude, frequency, duration) for moving individuals

(iii) A prototype tool for identifying individuals at risk among the population based on patterns of movement, hazard distributions and safety conditions to increase the efficiency in sampling (directed bio-monitoring and exposure measurements).

These benefits demonstrate how this approach could overcome the limitations of GIS based approaches of exposure assessment [24,49] if the necessary input data can be collected for the described agricultural production landscape.

There is evidence for policy relevance of the results presented. The assessment of health risks from pesticide exposure on an individual level allows for developing earmarked educational programs to reduce health risks of the exposure groups. The efficiency of such programs is likely to be higher since only relevant information about specific target groups is included in the program development.

### Limitations

The conceptual approach in its current state relies on simplified assumptions and interrelationships between the social and the environmental subsystem, as well as artificial input data. This was necessary since real data are lacking and the complexity had to be limited. The main objective, however, was to test feasibility of this approach for PEA and to fully understand the relevant mechanisms needed by developing a model prototype.

*First*, potential exposure is represented as a relative "degree" value because of a lack in exact information regarding pesticide use in the study area as well as a limited knowledge of the behavior of active ingredients for deposition and drift effects. Ideally calibration and validation of a PEA model such as ours will include the use of environmental and biological samples [56]. *Second*, we defined artificial patterns of movement as well as hypothetical weights for activities. More realistic patterns of movement would include evaluation of "real" daily patterns in agricultural production activities in the socio-cultural landscapes such as our study area, including an assessment of variability in repeatable patterns. We also propose to add variations in locations visited (e.g. home environment) and activities (e.g. person to person contact) to further elucidate potential exposure through the community. The definition of more reliable weights would require field measurements such as exposure values and GPS records of individuals, activity observations as well as health data. However, the developed mechanisms allow for the adjustment of the model once the required data are available. *Third*, temporal and spatial resolution, as well as the length of the model period are appropriate for this prototype demonstration, but always depend on the data available. Long term dynamics such as rotational shifts between cropping and pasture are not considered but would be important for an overall evaluation. *Fourth*, pesticide application was scheduled at the beginning of the day and crop fields for pesticide application were selected randomly. More realistic models would implement this event more accurately in time and incorporate field data to determine pesticide application activity. *Fifth*, the impact of transport of contaminant residuals at clothes and skin of the applicator on interpersonal exposure has not been included. The inclusion of this exposure pathway would add complexity to the model but would be an important part of a more realistic PEA in the study area.

### Follow-up research

To overcome these constraints, follow-up research has to focus mainly on the collection of data needed for calibration and validation of the model in the same area including exposure measurements and GPS records as well as interview data.

Because we used a dynamic model framework, we can readily incorporate behavioral and decision-making rules, which are influenced by economic issues, risk perception, the habits of everyday life [56], belief systems [1] or traditions [52,59] within a specific study area. Such rules can help to better identify individuals and groups at risk among the local population, including household members due to carry-home exposure by the worker. The goal is to evaluate regulative and intervention strategies to reduce health risk from pesticide exposure among farm-workers and other groups such as *residents *[50] or *children *[51,60] in similar agricultural production landscapes.

## Conclusion

In this paper a conceptual framework for assessing personalized exposure from pesticides in small-scale agricultural production landscapes of developing countries is presented and tested for feasibility. We conclude this feasibility study demonstrated our individual-based model prototype could differentiate exposure at the individual level across individual farm-workers with different patterns of movement by taking into account different safety scenarios and dynamic contaminant distributions. The use of the presented model framework allows for a more reliable determination of the individuals at risk among the population by breaking down the level of analysis to the individual level. It can thus provide a better understanding of the contribution of agricultural pesticide use to exposure in this type of agricultural landscape, which accounts for a substantial percentage of global agricultural production. There is potential to use this approach for evaluation of public health intervention strategies to reduce risks to the group of farm-workers and their families. Further research and data collection is needed to incorporate exposure measurements for validation of this approach.

## Competing interests

The authors declare that they have no competing interests.

## Authors' contributions

SL conceptualized the study, designed the methodology, developed and programmed the model, tested the model prototype and the scenarios, as well as wrote and revised this paper. CRB secured the spatial data layers of the study area, was involved in designing the conceptual idea and methodological steps and revised the paper. JRN was involved in the conceptual design and methodological development of the modeling approach, as well as in the strategic planning for scenario development and revised the paper. All authors read and approved the final manuscript.
